# Effect of Aggregate Type on Noise Characteristics and Emissions During the Crushing Process

**DOI:** 10.3390/ma19122646

**Published:** 2026-06-19

**Authors:** Paweł Ciężkowski, Damian Markuszewski, Mehmet Sait Şahinalp

**Affiliations:** 1 Division of Numerical Methods and Intelligent Structures, Faculty of Automotive and Construction Machinery Engineering, Warsaw University of Technology, Ludwika Narbutta 84 Street, 02-524 Warsaw, Poland; 2 Division of Machine Design Fundamentals, Faculty of Automotive and Construction Machinery Engineering, Warsaw University of Technology, Ludwika Narbutta 84 Street, 02-524 Warsaw, Poland; damian.markuszewski@pw.edu.pl; 3 Department of Geography, Harran University, Osmanbey Campus, 63050 Haliliye, Şanlıurfa, Turkey; sahinalp@harran.edu.tr

**Keywords:** jaw crusher, acoustics, mineral aggregates, degree of fragmentation, effect of material density, acoustic emission, the sound of materials being crushed

## Abstract

**Highlights:**

- Material structure governs fracture surfaces after jaw crushing.
- Porous materials form highly developed fracture morphologies.
- Crusher settings strongly control coarse product size distribution.
- Energy requirements were determined based on measurements of jaw force and displacement.
- By measuring the noise generated during shredding, it is possible to identify the type of material used.

**Abstract:**

In processes related to the treatment of mineral materials, the crushing stage determines the ability to obtain the required particle-size fraction. At the same time, it is an exceptionally energy-intensive step (accounting for about 5% of global electricity consumption) and one that generates significant environmental impacts, particularly in the form of high noise levels and considerable dust emissions. This study focuses on acoustic issues associated with the operation of crushers equipped with materials of varying hardness. Noise level measurements were carried out and then compared with the machines’ operational parameters, such as reduction ratio, throughput, energy consumption, and grain-size distribution. The results indicate that the properties of the processed material have a significant influence on noise emission during the crushing process. The study included various types of materials, such as pebble, basalt, and granite (feed size 16–22 mm), as well as lower-strength materials, including aerated concrete, recycled concrete, and ceramic materials (average particle size of approximately 50 mm), enabling a comparative analysis under controlled operating conditions. The measured noise levels ranged from front position 105.3 dB and side position 105.2 dB, depending on the material type, with the highest values observed for [hard material, e.g., recycled concrete and basalt] and the lowest for [weak material, e.g., aerated concrete]. The differences between extreme cases reached up to the top position 107.6 dB, indicating a strong relationship between material properties and acoustic emission. These findings highlight the importance of material selection in crushing processes and provide a useful reference for reducing noise impact and improving the environmental performance of industrial aggregate production.

## 1. Introduction

The crushing of brittle materials is one of the key stages in the technological processes involved in aggregate production [1,2,3,4,5,6]. Due to the increasing demand for high-quality construction materials and the growing emphasis on sustainable production, this issue has gained significant importance in both scientific research and industrial practice. Aggregates are widely used as materials for various structures in different fields of civil engineering [7], such as the production of concrete [7,8,9], asphalt layers [10], railway ballast, filters, and rock embankments. Various types of crushers, such as jaw crushers [11,12,13,14,15,16,17], cone crushers [18,19,20,21,22,23], and impact crushers [24,25,26,27,28,29,30,31,32], are used to reduce the size of brittle materials to the required fraction. A typical crushing plant consists of several crushers located at different stages of the crushing process and connected by belt conveyors [15,33,34].

Despite the widespread use of these machines, the physical phenomena occurring during crushing, especially dynamic interactions between material and machine components, are still not fully understood. During the operation of these devices, complex dynamic phenomena occur due to the interaction between the crusher’s working parts and the material being crushed, which affects the operation of the entire processing system and the resulting particle size distribution of the product [35,36].

An analysis of jaw crusher operation is crucial for improving efficiency, reducing energy consumption, and extending the service life of wear-prone components, particularly the jaws, that is, the fixed and movable jaws [37,38,39,40]. Parameters such as the grip angle, shaft rotation speed, feed opening size, and material properties significantly influence the efficiency of the crushing process carried out by this type of crusher [17,41]. However, existing studies typically focus on selected parameters and do not provide a comprehensive analysis of their combined influence on process performance and environmental effects.

One of the major operational issues is the generation of vibrations and noise emissions [42,43] which can affect both the durability of the crusher’s structural components and the working conditions of machine operators, as well as the environmental impact of the production line. Among the many industrial hazards, vibration and noise hazards are among the most dangerous. These are, in fact, hazards present in all environments. Noise and vibrations significantly increase the likelihood of health issues and workplace accidents [44]. The intensity of these phenomena depends on many factors, including the type of crushing machine, the mechanical properties of the material, the operating parameters of the equipment, and the degree of wear on the working components [40,45]. Despite their importance, vibration and noise are often treated as secondary effects rather than key parameters in the analysis of crushing processes.

Given the growing requirements for workplace safety and environmental protection, there is a growing need for systematic identification and analysis of vibration and noise sources in crushing processes. Understanding the mechanisms behind these phenomena can contribute to optimizing the operation of crushing machines, increasing their service life, and reducing their negative impact on the environment.

The efficiency of the crushing process directly affects energy consumption [46,47,48,49,50,51,52,53], plant productivity, and the quality of the final product. Therefore, it is crucial to develop methods that enable real-time monitoring of the operating parameters of crushing machines and assessment of the technical condition of the equipment. In recent years, a clear trend toward the development of advanced monitoring and diagnostic systems based on real-time data has been observed.

In recent years, increasing attention has been paid to the use of diagnostic methods based on the analysis of dynamic signals generated during machine operation [54]. The most commonly used methods are those based on measurements of vibration and acoustic emissions [55,56]. These signals contain information regarding both the technical condition of machine components and the course of the technological process itself. However, their application is often limited by the complex and non-stationary nature of the signals.

In the case of crushers, however, vibration signals are complex in nature, resulting from the impulsive interaction of feed grains with the crushing plates [57]. An additional source of interference is the technological processes occurring in the crushing chamber, such as grain collisions or their movement within the working chamber [58,59,60,61,62]. This results in the generation of non-stationary signals with high amplitude variability, which significantly complicates their interpretation and requires the use of advanced signal processing methods. Consequently, classical signal analysis approaches are often insufficient.

Acoustic emission signals can serve as an alternative or supplementary source of diagnostic information [63,64]. The phenomenon of acoustic emission involves the generation of elastic waves in a material as a result of processes such as deformation, fracture, or friction between system components. In crushing processes, these signals are directly related to material breakage, which makes them particularly valuable for analyzing process mechanisms.

The development of modern process monitoring systems has led to growing interest in integrating measurements of vibration, acoustic emissions, and other diagnostic signals into machine monitoring systems [65,66,67,68,69,70]. This multi-sensor integration approach represents an important development trend in machine diagnostics [57,60,71,72]. A multi-method approach enables a more reliable assessment of the technical condition of equipment and a more effective identification of changes occurring in the technological process [55,57,60,73,74].

In recent years, there has been a dynamic development of machine learning (ML) methods that are increasingly being applied in monitoring and diagnosing machines used in mineral processing [75,76,77]. These methods enable the extraction of complex relationships from experimental data that are difficult to capture using classical analytical approaches.

Despite these developments, several important research gaps remain. To better illustrate the limitations of existing research, a summary of selected studies is presented in Table 1. The table highlights the main research scopes, key findings, and identified gaps, particularly in relation to the insufficient integration of process parameters, environmental aspects, and dynamic signal analysis.

As shown in Table 1, most existing studies focus on selected aspects of the crushing process, such as energy consumption, particle size distribution, or environmental impact. However, there is a clear lack of comprehensive approaches that combine these aspects with the analysis of vibration and acoustic signals. This confirms the need for further research aimed at integrating physical process parameters with advanced diagnostic methods.

Therefore, although data-driven approaches are increasingly important, physically interpretable models remain essential for understanding fundamental relationships between operating parameters (e.g., CSS) and process outcomes.

Consequently, the objective of this study is to develop an approach that combines the analysis of selected physical quantities, noise, forces, and process energy, with interpretable modeling of the crushing process. The proposed methodology enables a more comprehensive characterization of the relationships between process parameters and particle size distribution. This study addresses the identified research gaps by integrating process-related and environmental factors into a unified analytical framework.

## 2. Materials and Methods

### 2.1. Raw Material

The study utilized five different feed materials that differed in their physical and mechanical properties, as well as their internal structure. The use of materials with varying strength parameters made it possible to analyze the impact of feed characteristics on the crushing process and on the characteristics of the recorded vibration and noise signals.

One of the materials used was a pebble with a particle density of 2.85 g/cm^3^, characterized by a compact structure. During the experiments, the material exhibited limited fragmentation compared to the other materials tested. Due to the rounded shape of the grains, this material presented an interesting case in the analysis of the crushing process, as its geometry influences the manner of contact with the crusher’s grinding plates and the course of the grinding process. The pebble aggregate used in the study was supplied by Wanopol, a company based in Płońsk.

Another material studied was aerated concrete with a particle density of 0.6 g/cm^3^ [83], which is characterized by high porosity. The use of this material allowed for an analysis of the crushing process of low-density materials exhibiting a porous internal structure and a high degree of fragmentation during testing. According to the manufacturer’s data, this concrete complies with the EN 771-4:2011+A1:2015 standard [84].

Basalt with a particle density of 2.9 g/cm^3^ was also used in the studies; it is an igneous rock with high hardness and high mechanical strength. This material is commonly used as aggregate in construction and road engineering, which is why analyzing its behavior during the crushing process is of significant practical importance. The basalt aggregate used in the study was supplied by Wanopol in Płońsk, Poland.

Another material was granite [85] with a particle density of 2.63 g/cm^3^, characterized by a very compact crystalline structure and high resistance to compression and abrasion. Granite is a typical natural raw material in aggregate production, so its use allowed for the evaluation of the behavior of hard, homogeneous rocks during the crushing process. The granite aggregate used in the study was obtained from SOBEX Sp. z o.o. (Drezdenko, Poland).

The penultimate material analyzed was recycled ceramic with a particle density of 2.3 g/cm^3^, which is used in the production of arc chambers and quenching chambers for SU contactors [86]. This material is characterized by a brittle structure and irregular grain shape. The inclusion of recycled ceramics in the study was intended to assess the feasibility of monitoring the crushing process of secondary materials, which are increasingly used as raw materials in a circular economy. The ceramic aggregate used in the study was obtained from the Łukasiewicz Institute of Ceramics and Building Materials in Warsaw, Poland.

**Figure 1:**
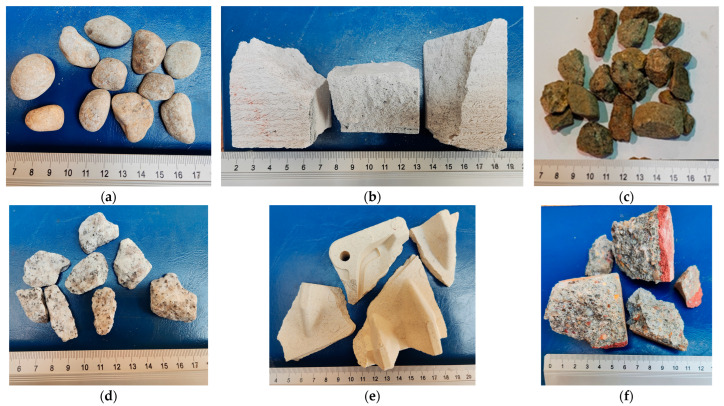
Shapes of feedstock particles: (**a**) pebble; (**b**) aerated concrete; (**c**) basalt; (**d**) granite; (**e**) ceramics; (**f**) recycled concrete.

The last material tested was aggregate obtained from recycled paving stones, with a particle density of 2.4 g/cm^3^. The source material consisted of two-layer paving blocks, comprising a pigmented surface layer and a concrete base layer. Consequently, the resulting aggregate contained particles from both layers (Figure 1f). The concrete aggregate used in the study was supplied by Wanopol, a company based in Płońsk.

The use of materials with diverse properties enabled a comparison of the crushing process and an assessment of the influence of feed characteristics on the vibration and noise signal parameters recorded during crusher operation. The grain shapes of the feed for all materials used in the study are shown in Figure 1.

Table 2 presents the grain sizes used in the tests for various materials. For basalt, granite, and cobblestone, the grain size distribution was determined using sieve analysis, while for ceramics, recycled concrete and aerated concrete, the average grain size was estimated by measuring the grains with a caliper. The second column of the table lists the mass of the prepared feed samples for each material, allowing for a quantitative comparison of the raw materials used in the experiment.

### 2.2. Research Station

**Figure 2:**
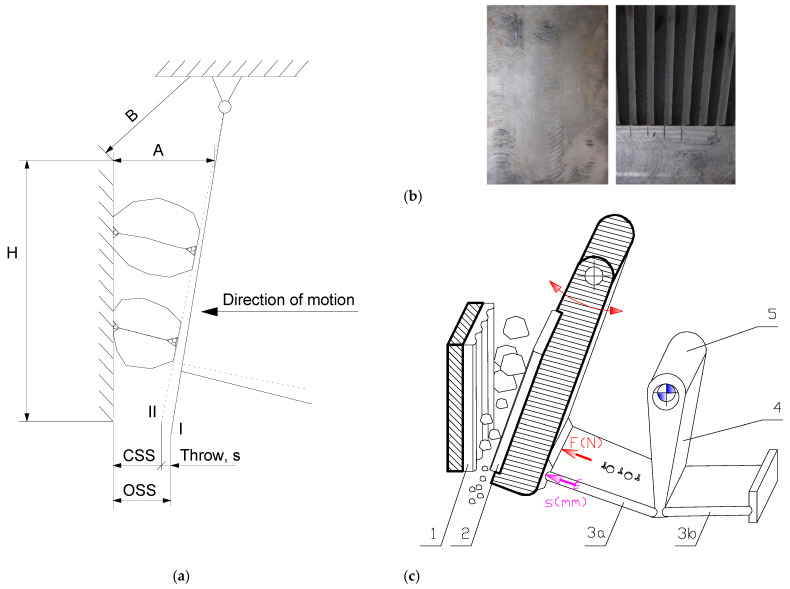
Laboratory jaw crusher: (**a**) Measurement of the outlet slot: I—jaw crusher in open side discharge setting—OSS, II—jaw crusher in closed side discharge setting—CSS (adapted from [86]); (**b**) Crushing plate profile; (**c**) F—force measurement system, s—moving jaw displacement measurement system (adapted from [87] (modified)), (1) fixed jaw, (2) moving jaw, (3a, b) front end rear toggle, (4) pitman, (5) eccentric shaft.

The crushing process was studied using a Blake-type laboratory jaw crusher equipped with an eccentric mechanism that ensures periodic movement of the movable jaw relative to the fixed jaw. The material crushing process took place in the crushing chamber between plates of different shapes, between which the feed grains were crushed. The fixed plate had a smooth surface, while the movable plate had variable pitch and notch height (Figure 2b). The test crusher has the following technical parameters: crusher inlet gap dimensions A × B = 100 mm × 200 mm, outlet gap adopted in this study CCS = 5 mm, working chamber height H = 250 mm, movable jaw stroke s ≈ 6 mm, crusher drive shaft speed n = 388 rpm, and rated motor power N = 4 kW. The test stand is equipped with specialized recording and control equipment [87].

Material with a specific particle size range was fed into the crusher through the upper feed opening, while the crushed product exited the crushing chamber through an outlet slot with an adjustable width. The operating parameters of the device were maintained at a constant level throughout each series of measurements.

**Figure 3:**
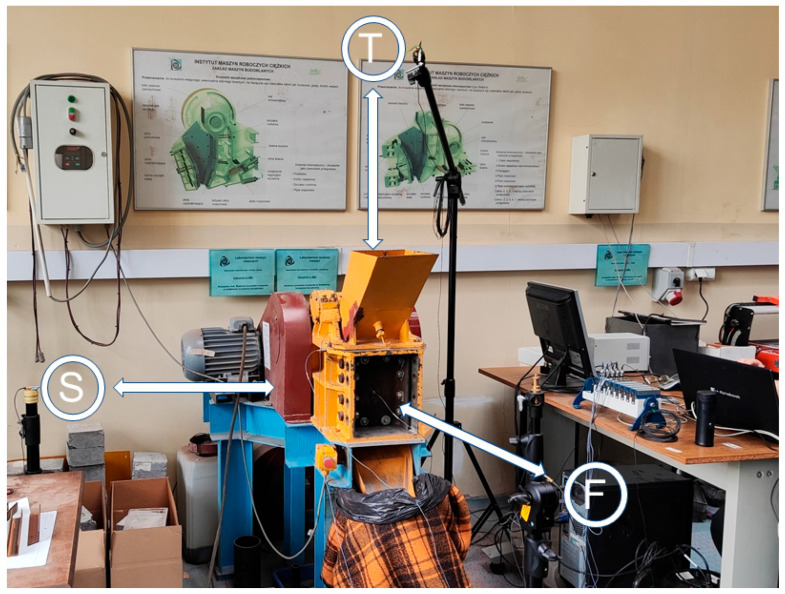
Placement of measurement sensors on the crusher in the experimental setup—noise level measurement: F—front, S—side, T—top.

To record the dynamic signals generated during the crusher’s operation, the test stand was equipped with measurement equipment capable of measuring noise [88] (Figure 3).

The Blake-type jaw crusher used in this study reflects the design solutions employed in industrial equipment, and the test conditions and operating parameters adopted correspond to those encountered in actual operation. At the same time, it should be noted that despite efforts to replicate industrial conditions, laboratory-scale studies may not fully reflect all aspects of the crusher’s operation under real-world conditions, due to process scaling limitations, simplifications in operating conditions, and the controlled nature of laboratory testing [89].

Signals from the Bruel & Kjaer 4958 microphones placed 1 m from the crusher in three directions were transmitted to a data acquisition system consisting of an eDAQ-9178 cassette and a National Instruments NI 9234 measurement card, where they were digitized and recorded as time-domain waveforms in NI LabVIEW 2015 software. Data recording was performed at a sampling rate of 25,000 Hz, enabling the analysis of dynamic phenomena occurring during the crushing process.

The microphone was positioned to capture acoustic signals associated with the crushing process while minimizing the impact of ambient noise.

The recorded noise signals were subjected to further analysis using MATLAB 2024b software, incorporating signal processing methods to identify parameters characterizing the crushing process.

In parallel with the noise measurements, mechanical parameters directly related to the crusher’s operation were also measured. In particular, the crushing force and the displacement of the movable jaw were recorded, which enabled a more accurate characterization of the crushing process.

The crushing force was measured using a force sensor installed in the crusher’s structural assembly, in a location that allowed for the recording of loads generated during the crushing of material in the crushing chamber. The recorded force values reflected changes in the load on the crushing system during the machine’s operating cycle.

At the same time, the displacement of the movable jaw was measured using a suitable displacement sensor mounted near the eccentric mechanism that drives the jaw. Recording the displacement made it possible to determine the jaw’s motion over time and correlate it with changes in crushing force.

The simultaneous recording of vibration, noise, crushing force, and jaw displacement signals allowed for a comprehensive analysis of the crushing process. The comparison of these parameters enabled the identification of the relationship between the load on the crushing system and the generated dynamic signals, which forms the basis for evaluating the potential use of vibration and noise signals to monitor the crushing process.

All measurement signals recorded during the tests, including vibration accelerations and changes in sound pressure level, were recorded using a common data acquisition system. The use of a single measurement system enabled the temporal synchronization of most of the recorded signals.

Synchronization of the measurements was essential to ensure the ability to directly compare changes in individual parameters over time. This made it possible to correlate characteristic changes in vibration and noise signals with the crusher’s operating cycle, as well as with the instantaneous values of crushing force and the displacement of the movable jaw.

The recorded data were saved as a time series, which enabled an analysis of the relationships between the various parameters of the crushing process. In particular, the moments at which maximum crushing forces occurred were analyzed, along with the corresponding changes in vibration amplitude and sound pressure level. This approach allowed for the identification of characteristic features of dynamic signals related to the intensity of the material crushing process.

The achieved signal synchronization also enabled time- and frequency-domain analysis, as well as an assessment of the correlation between the mechanical parameters of the crushing process and the diagnostic signals recorded on the crusher body.

### 2.3. Measurement Methods

#### 2.3.1. Measurement of Grinding Process Parameters

Measuring the parameters of the crushing process in a jaw crusher involves recording both input values and operating parameters, as well as the properties of the final product. The basic process parameters include the particle size distribution of the feed and the product [90,91,92], crushing capacity [93], degree of reduction, and the energy parameters of the process [12].

The analysis of feed and product particle size was conducted in accordance with applicable standards, using sieve methods that allow for the determination of particle size distribution [94]. On this basis, characteristic parameters such as the d_50_ [1] and d_80_, as well as the degree of fineness, defined as the ratio of the characteristic dimensions of the feedstock and the product.

During the study, the crusher’s operating parameters—such as the forces and loads acting on the machine’s working components [87]—were recorded, which allowed for an assessment of the operating conditions and the characteristics of the crushing process. Another important measurement was the analysis of energy consumption, determined based on the measurement of force in the spreader plate and the displacement of the movable jaw, which made it possible to determine the energy efficiency of the crushing process [95,96]. The detailed measurement methodology and the research apparatus used are presented in the authors’ earlier works [86,87]. Crushing force was measured by using the crusher’s front spreader plate to act as a dynamometer. For this purpose, two holes were drilled in the spreader plate, symmetrically positioned relative to the x and y axes, into which strain gauges were mounted. The arrangement of the strain gauges on the spreader plate, connected in a Wheatstone bridge, ensures the measurement of longitudinal force while simultaneously eliminating effects resulting from bending, torsion, and temperature changes.

In the literature, the most common measurements are those of the power consumed by the crusher drive and the operating time of the machine [93,97,98].

#### 2.3.2. Noise Measurement

To monitor the crushing process, measurements were taken of the noise generated during the crusher’s operation. Recording dynamic signals allows for the analysis of phenomena occurring in the crushing chamber and an assessment of how the machine’s operating parameters affect the technological process.

Noise measurements were taken using microphones mounted 1 m away from the crusher body. The sensors were installed near structural joints that bear the loads associated with the crushing process, which allows for the recording of noise generated both by the operation of the crushing mechanism and by the impact of the crushed material on the machine’s working components.

Acoustic signals were recorded as changes in sound pressure over time. Measurement data were collected at a sampling rate of 25,000 Hz, ensuring accurate recording of the impulsive dynamic [99,100,101] characteristic of the crushing process, i.e., up to 250,000 Hz associated with the Nyquist frequency. The recorded signals were then analyzed in the time and frequency domains to identify diagnostic features related to changes in process parameters.

The recorded acoustic signals were analyzed to determine the sound pressure level and the spectral characteristics of the noise. Analysis of the acoustic signals enables the identification of changes in the intensity of the crushing process and the assessment of the impact of the machine’s operating conditions on noise emission.

### 2.4. Microscopic Observations

The microstructure was examined using a Leica M205A stereoscopic microscope (Wetzlar, Germany) [102]. The analysis focused on grains retained on a sieve with a mesh size of 8 mm, with particular attention paid to the morphology of the fracture surface. Observations were made at magnifications of up to ×11.7 [83].

### 2.5. Basic Parameters of the Crushing Process and Their Calculation

**Figure 4:**
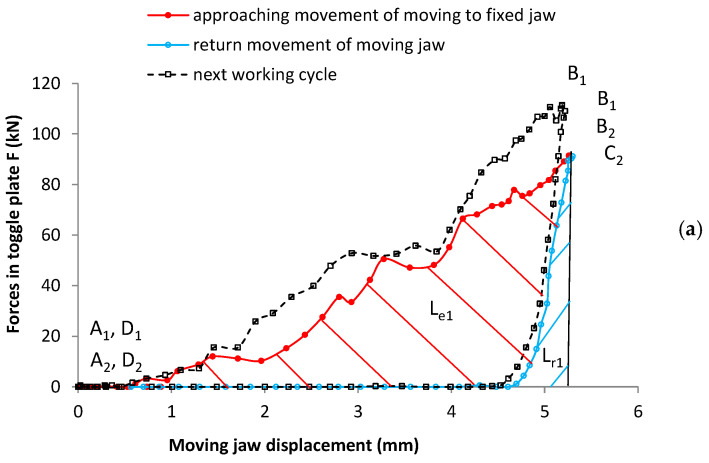

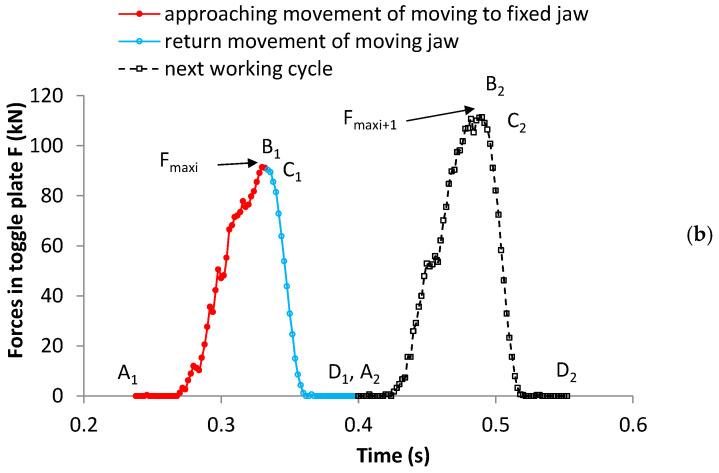
Selected two work cycles of basalt crushing between plates of different shapes: (**a**) force vs. displacement diagram and (**b**) force vs. time diagram.

Calculating the actual parameters of the crushing process is of great importance in terms of total production costs. Furthermore, it is necessary to determine the load—which depends on the material being crushed—in order to select the appropriate crushing process for the material in question. During the crushing process, forces undergo cyclic changes, and their values vary stochastically depending on the distribution of material within the crusher chamber. To compare crushing results using specific materials, a set of indices has been introduced. These indices relate to forces, energy, and productivity. They are as follows:

- *F_peak_*—maximum crushing force defined as the highest value recorded in one sample of the crushing process;
- *L_ei_*—effective crushing energy in one cycle is determined as follows:

(1) $$L_{ei}=\oint Fds=\sum_{j=1}^{n}\left(\frac{1}{2}\cdot \left(F_{j}+F_{j-1}\right)\cdot \left(s_{j}-s_{j-1}\right)\right),\;\;j=1,........,n$$

where *F_j_*, *s_j_*—forces and displacements measured in *n* points in one cycle of operation in Figure 4, or as a sum of crushing energy in one cycle, energy returned to the system:

(2) $$L_{ei}=L_{ci}+L_{ri}=\int_{A}^{C}Fds+\int_{C}^{D}Fds$$

Figure 4 shows the selected two consecutive cycles of the basalt crushing between plates of different shapes (Figure 4b—enlarged diagram in the time period from 0.238 s to 0.552 s). These charts were created by recording n measurement points (marked symbols). The crushing process takes place periodically. With the approach of moving to the fixed jaw thrust of the jaws increases, force increases unevenly, sometimes experiencing a little decrease associated with the local rock crushing process. The highest values of toggle plate thrust exist near the point of maximum approach of the moving jaw to the fixed jaw (marked points B_1_ and B_2_ in Figure 4a,b). The point C_1_ and C_2_ starts a return movement of the jaw during the rapid reduction in the thrust force. In this study, the displacements of the moving jaw reach 5.3 mm (points D_1_ and D_2_). The variable value of the moving jaw displacement is associated with the flexibility of the crusher. During the return movement, the recovery of elastic energy accumulated in the brittle material and in the crusher, which, through the toggle plates, is supplied to the flywheel of the crusher.

- *L_ci_*—crushing energy in one cycle Figure 4a

(3) $$L_{ci}=\int_{A}^{C}Fds=\sum_{l=1}^{k}\left(\frac{1}{2}\cdot \left(F_{l}+F_{l-1}\right)\cdot \left(s_{l}-s_{l-1}\right)\right),\;\;l=1,........,k$$

where *F_l_*, *s_l_*—forces and displacements measured in *n* points in one cycle of operation on line A–C in Figure 4a.

- *L_ri_*—crushing energy returned to the system in one cycle, Figure 4a

(4) $$L_{ri}=\int_{C}^{D}Fds$$

- *L_e_*—effective crushing energy is equal to the sum of energy obtained in subsequent crushing cycles

(5) $$L_{e}=\sum_{i=1}^{k}L_{ei},\;i=1,........,k$$

- *L_s_*—specific energy is the ratio of the effective energy *L_e_* recorded in a given specimen to its mass *m*:

(6) $$L_{s}=\frac{L_{e}}{m}$$

- *W_t_*—technical performance—feed mass to crushing time ratio:

(7) $$W_{t}=\frac{m}{t}$$

## 3. Results

### 3.1. Particle Size Distribution and Morphology

The particle size distribution of the crushed product was determined using sieve analysis. The resulting particle size distribution is shown as a particle size curve (Figure 4).

**Figure 5:**
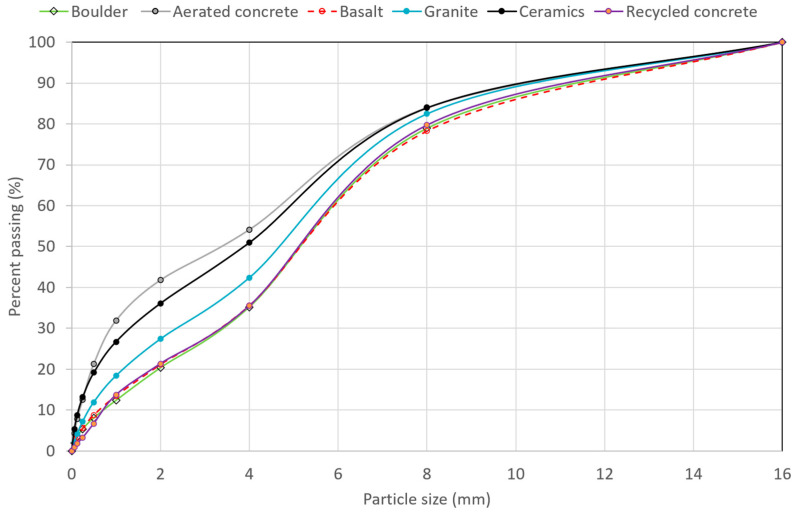
Particle size distribution of crushing product determined by sieve analysis.

Figure 5 shows the crushed product obtained from the crushing process for five materials: (a) cobblestone, (b) aerated concrete, (c) basalt, (d) granite, (e) ceramic materials, and (f) recycled concrete. The variation in grain morphology (shape, surface area) and particle size distribution reflects different fracture and propagation mechanisms, determined by the mechanical properties and internal structure of the raw materials (brittleness, porosity, anisotropy). These observations are consistent with literature reports on the influence of material characteristics on the course of the grinding process and the resulting product particle size distribution [89].

**Figure 6:**
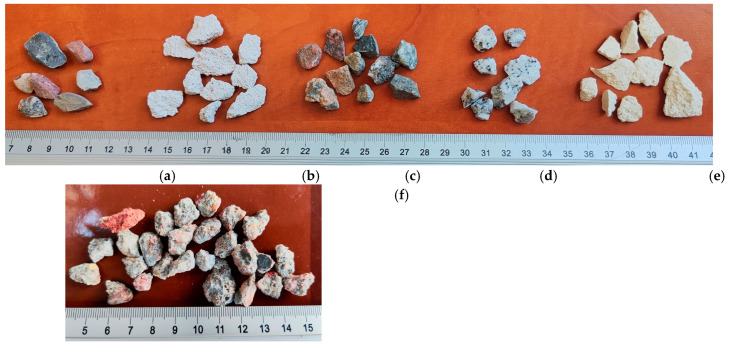
Grain size greater than 8 mm: (**a**) pebble; (**b**) aerated concrete; (**c**) basalt; (**d**) granite; (**e**) ceramics; (**f**) recycled concrete.

**Figure 7:**
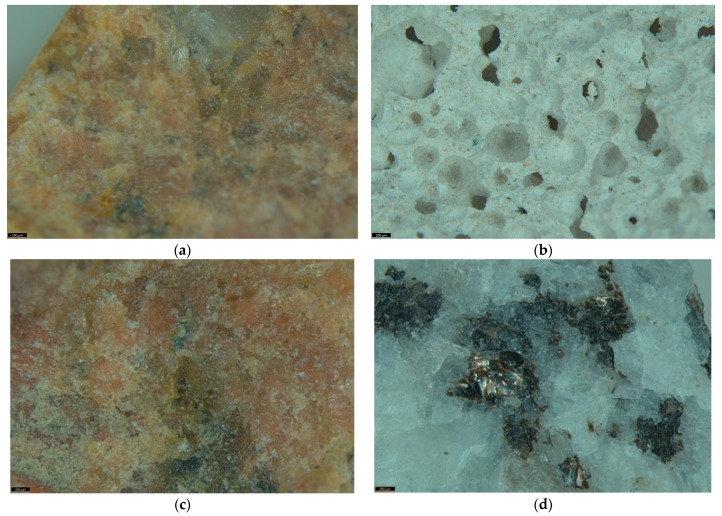

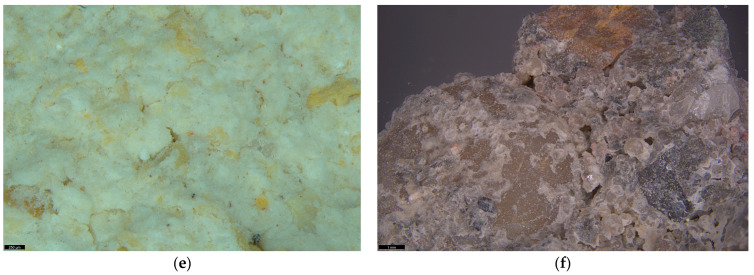
Particle sizes of the materials after grinding: (**a**) pebble; (**b**) aerated concrete; (**c**) basalt; (**d**) granite; (**e**) ceramics; (**f**) recycled concrete.

Figure 6 shows the surface of sample grains larger than 8 mm: river pebbles, aerated concrete, ceramics, granite, basalt, and recycled concrete, obtained after the crushing process. The scale bar in all panels corresponds to 0.250 µm, except for panel 6f, where it corresponds to 1 mm (Figure 7). Analysis of the surface morphology of the grains after crushing reveals significant differences resulting from the structural composition of the tested materials. Aerated concrete is characterized by a developed, porous fracture [83], whereas rock materials, such as granite and basalt, exhibit more compact and irregular fracture surfaces of a brittle nature. Ceramics are characterized by a relatively homogeneous fracture surface with local discontinuities, while river pebbles exhibit surfaces resulting from fracturing, differing from the originally smoothed form obtained during transport processes. The observed differences in the nature of the fracture surfaces may influence the grinding mechanism and the energy consumption of the crushing process.

### 3.2. Sample Waveforms of Recorded Variables

**Figure 8:**
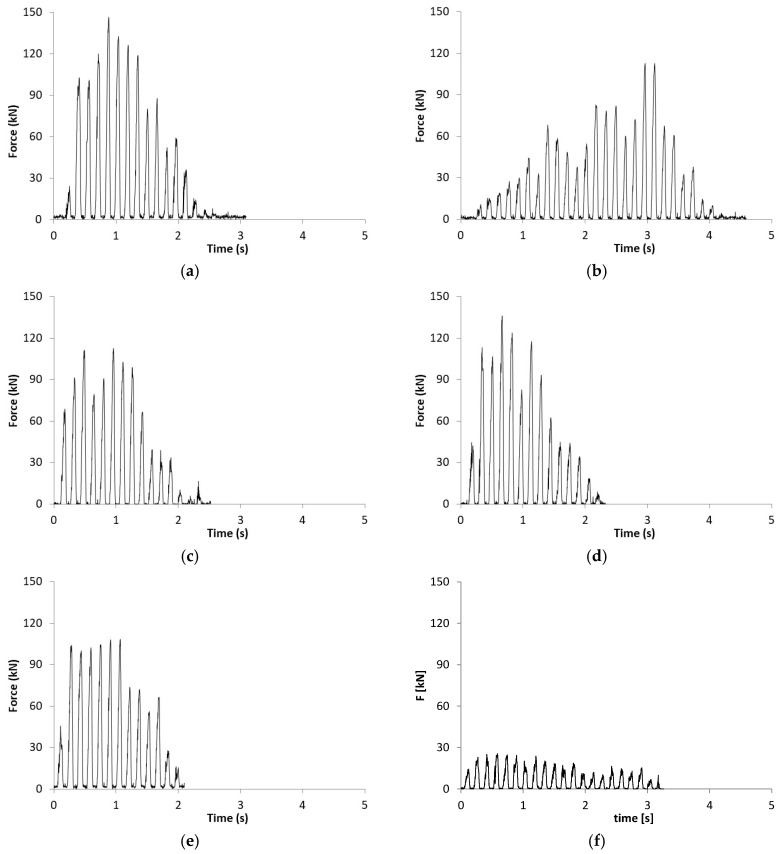
Changes in normal force as a function of time (**a**) pebble; (**b**) aerated concrete; (**c**) basalt; (**d**) granite; (**e**) ceramics; (**f**) recycled concrete.

**Figure 9:**
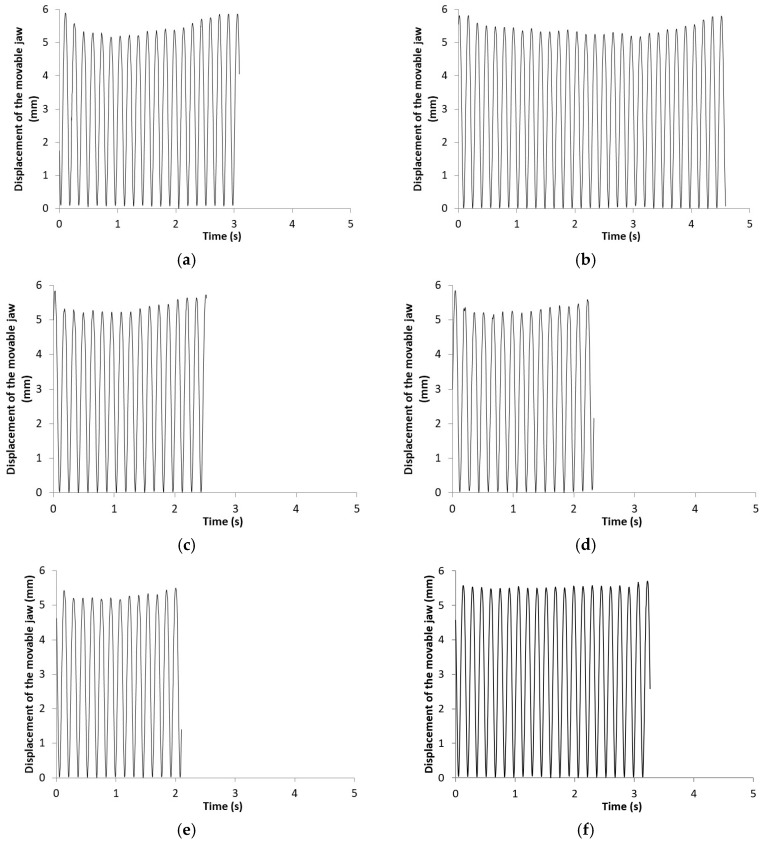
The relationship between the displacement of the movable jaw and time: (**a**) pebble; (**b**) aerated concrete; (**c**) basalt; (**d**) granite; (**e**) ceramics; (**f**) recycled concrete.

Examples of force-time curves for the front plate of a jaw crusher are shown in Figure 8. Figure 9 shows the displacement of the movable jaw as a function of time. It can be observed that in extreme cases, when the force reaches its maximum values (Figure 7), the displacement of the jaw decreases.

### 3.3. Analysis of Indicator Values

To comparatively evaluate the effects of grinding different materials, a set of indicators was used to characterize the forces, energy consumption, efficiency, and degree of grinding (Table 3). The detailed methodology for determining these indicators is described in [86,95,96].

The efficiency of the crushing process depends on both the feed particle size and the material properties. For river pebbles, basalt, and granite with uniform particle sizes, similar efficiency values were obtained. In the case of aerated concrete, a significant drop in efficiency was observed, which was associated with periodic jamming of the grains between the crusher jaws and disruption of the process continuity. In contrast, technical ceramics, despite the larger size of the feed material, exhibited the highest throughput, indicating a significant influence of their properties (e.g., brittleness) on the course of the grinding process.

The specific energy consumption values obtained for the grinding process of the analyzed materials range from 0.000220 to 0.000570 kWh, indicating a relatively low but clearly varying energy demand depending on the type of material.

The lowest energy consumption was obtained for recycled concrete (0.000220 kWh), while the highest was for cobblestone (0.000570 kWh). The remaining materials (aerated concrete, granite, and ceramic materials) showed intermediate and very similar values (approx. 0.00049 kWh), suggesting their similar susceptibility to crushing under the experimental conditions used.

According to the literature on crushing mechanics, specific energy consumption depends heavily on the physical and mechanical properties of the material—in particular its brittleness, internal structure, and fracture resistance—rather than solely on the feed particle size [89]. More brittle materials, with lower resistance to crack initiation and propagation, require less energy input, which is confirmed by the low energy consumption of basalt in our own studies.

At the same time, the observed differences between materials with similar energy values (aerated concrete, granite, ceramics) may result from the competing influence of two factors: on the one hand, feed particle size, and on the other, the differentiated microstructure of the materials, which is also emphasized in experimental studies on crushing in jaw crushers [11].

These results therefore confirm that the energy intensity of the crushing process is not unambiguously determined by the type of material in a macroscopic sense (e.g., rock vs. aerated concrete), but rather results from its actual structural and mechanical properties, which can cause significant differences even under similar process conditions.

The obtained values of specific energy consumption (0.44–1.14 kWh/Mg) are consistent with literature data on the crushing process in jaw crushers, which indicate that the energy required for crushing strongly depends on the material’s physical -mechanical properties, such as compressive strength, brittleness, and internal structure. Experimental studies show that differences in crushing energy result primarily from material characteristics, rather than solely from process parameters or feed size [97].

Similar relationships have also been confirmed in studies on energy consumption in jaw crushers, where it was shown that materials with higher mechanical strength require significantly more energy, while brittle materials are characterized by lower energy requirements [98]. Furthermore, the literature indicates that classical energy approaches (e.g., Bond’s theory) do not always fully describe actual energy consumption, as the actual crushing process depends on complex fracture and material–tool contact phenomena [103].

The obtained results thus confirm that the energy intensity of the crushing process is determined mainly by material properties, which is consistent with observations presented in the literature on crushing mechanics.

The obtained values of specific crushing time (1.161–2.544 h/Mg) indicate a clear variation in process efficiency depending on the type of material. The shortest crushing times were obtained for ceramic materials (1.161 h/Mg) and granite (1.294 h/Mg), which indicates their high susceptibility to fragmentation under the analyzed conditions. Slightly higher values for basalt (1.389 h/Mg) and pumice (1.667 h/Mg) indicate greater resistance of these materials to the crushing process.

The highest value of the parameter was recorded for aerated concrete (2.544 h/Mg), indicating the lowest process efficiency. This may result from its porous and heterogeneous structure, which promotes material jamming between the crusher jaws and disruptions in process continuity.

The results obtained confirm that the specific crushing time depends not only on the aggregate size but, above all, on material properties such as structure, brittleness, and homogeneity, which determine the course of the process and its efficiency.

The variation in the i_80_ index values (2.54–6.56) stems primarily from differences in feed particle size, as a larger d_80_ value (~50 mm) was used for aerated concrete and ceramic materials than for the other materials (~20.8 mm), which directly affects the degree of fragmentation achieved. Consequently, the comparison of i_80_ values among the analyzed materials is limited in scope and should not be treated as a direct measure of the influence of material properties.

### 3.4. Sound Pressure Characteristics

During the experiment, noise was recorded using a Bruel & Kjaer 4958 array microphone. The key specifications of this microphone are as follows:

- Frequency range: 10 Hz–20 kHz;
- Dynamic range: 28 dB(A) to 140 dB SPL;
- Technology: TEDS;
- Power supply: CCLD/IEPE.

**Figure 10:**
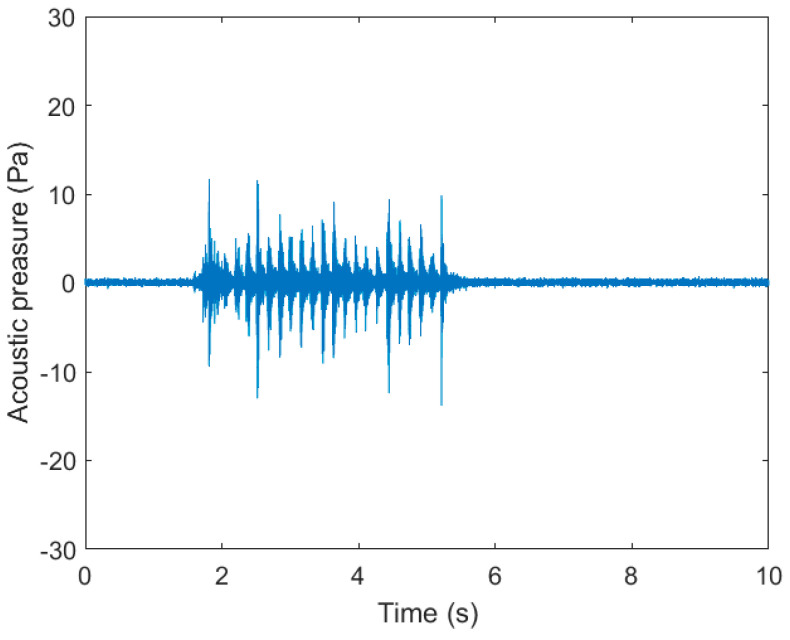
Recycled concrete.

**Figure 11:**
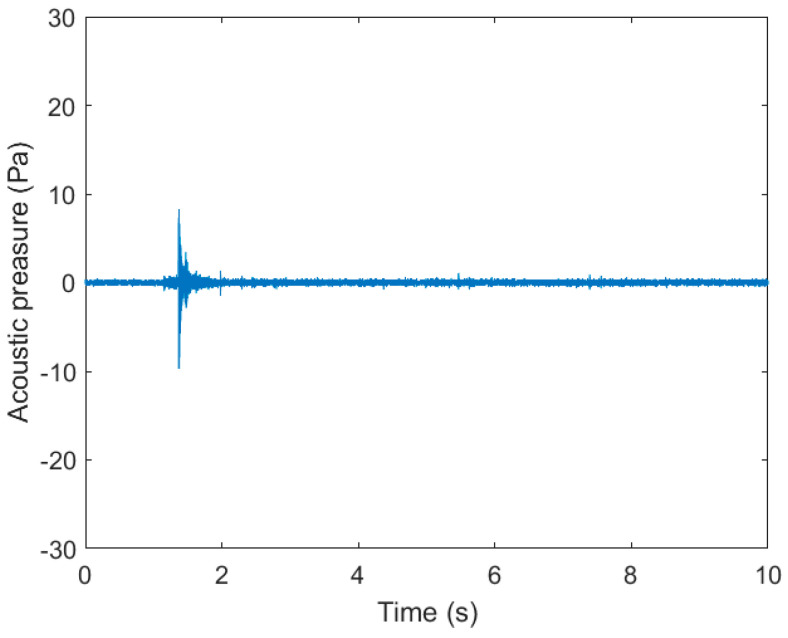
Aerated concrete.

**Figure 12:**
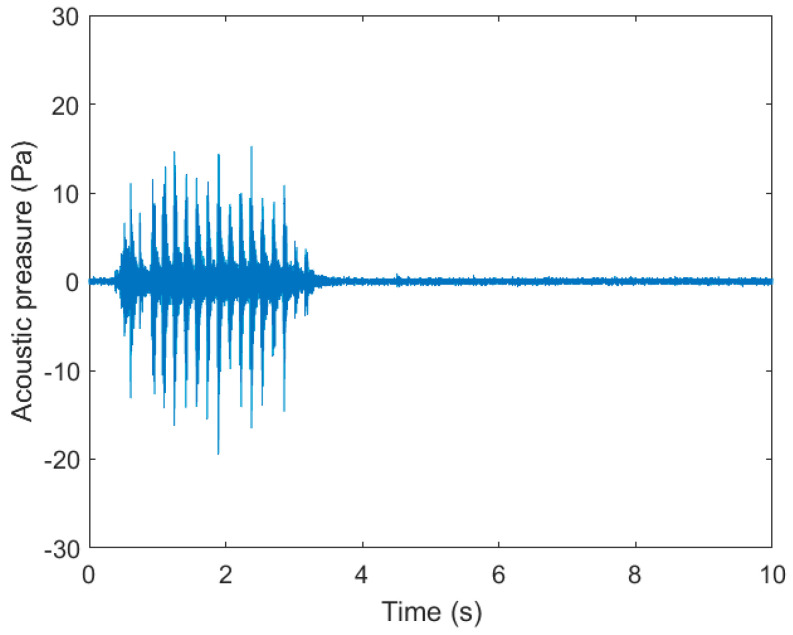
Granite.

**Figure 13:**
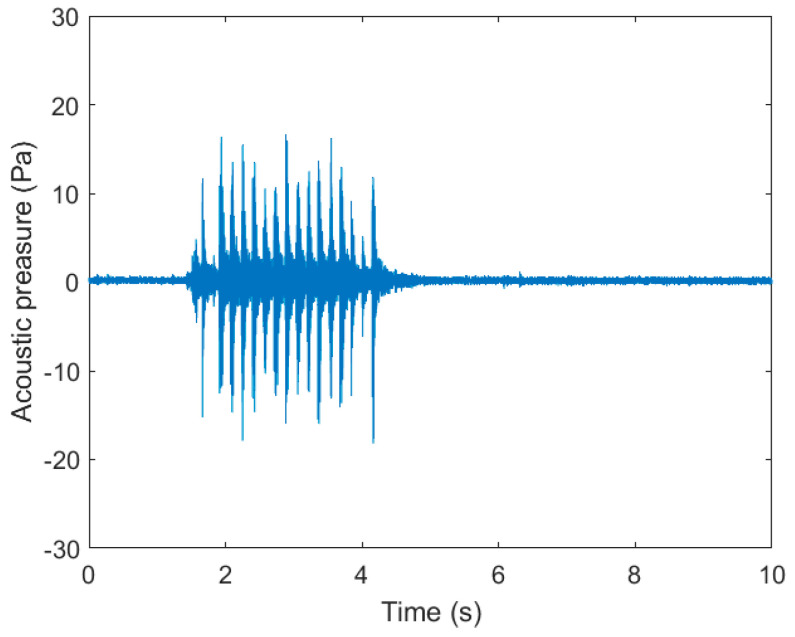
Basalt.

**Figure 14:**
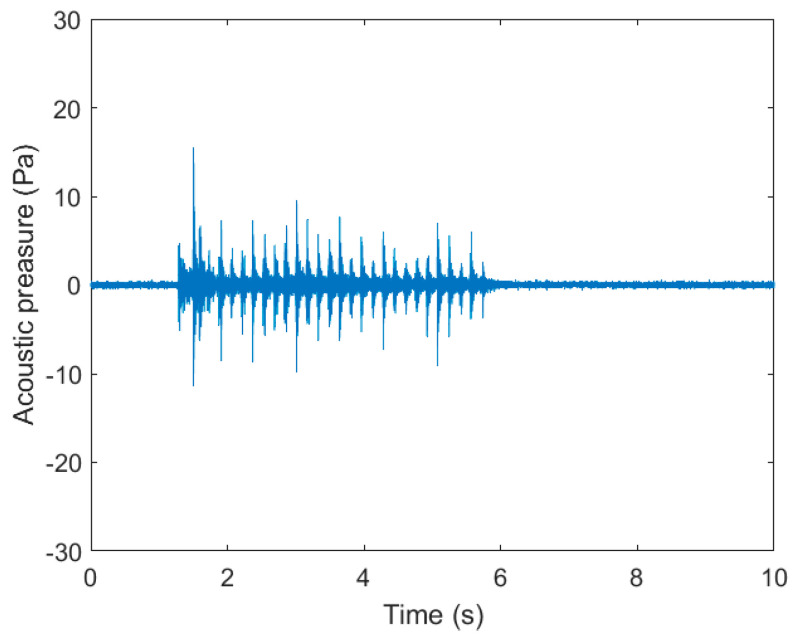
Ceramics.

**Figure 15:**
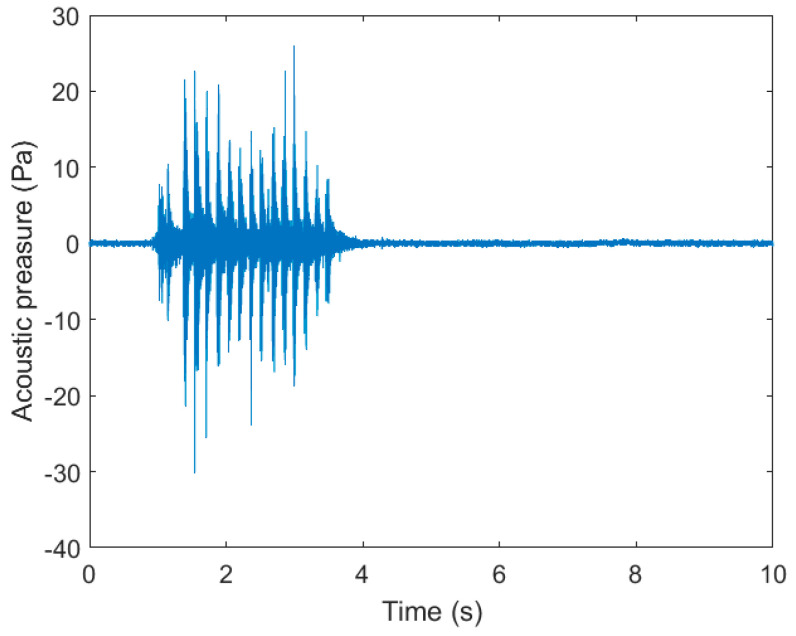
Pebble.

The advantages of this microphone include excellent phase preservation, precise mapping of the acoustic field in the immediate vicinity of the object, and minimal interference in the airflow. Figure 10, Figure 11, Figure 12, Figure 13, Figure 14 and Figure 15 show the signals in the time domain. They reveal several jaw strikes and the successive stages of feed grinding.

**Figure 16:**
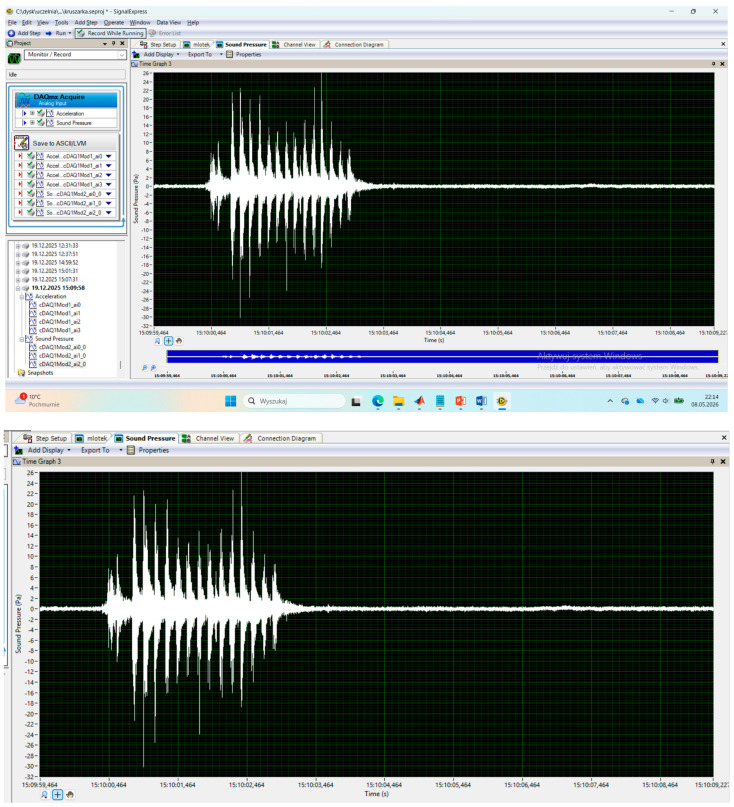
SignalExpress 2015 measurement system.

Data acquisition was performed using National Instruments’ SignalExpress 2015 system (Figure 16).

Voltage values were recorded in millivolts (mV), then divided by the microphone’s sensitivity of 11.2 mV/Pa to obtain pressure in Pascals, and further converted, taking into account the reference pressure level, to sound pressure level in dB (1 Pa = 94 dB SPL).

To analyze the noise structure, a spectral analysis of the signal was performed in third-octave bands. The width of such a band is 1/3 of an octave. This analysis is performed to accurately identify noise sources. As part of the analysis, instead of providing a general noise level, the spectrum was divided into 26 frequency intervals—bands of constant width. The resulting spectrum shows the distribution of signal (noise) energy as a function of frequency. Each bar represents the sound pressure level in decibels (dB) at a specific center frequency.

(8) $$f_{o}=\sqrt{f_{d}-f_{g}}$$

where

$f_{o}$—center frequency,

$f_{d}$—low frequency,

$f_{g}$—upper frequency.

The upper and lower frequencies are determined based on the following relationship:

(9) $$f_{d}=f_{o}\cdot 2^{-1/6}$$

(10) $$f_{g}=f_{o}\cdot 2^{1/6}$$

And the bandwidth:

$$f_{g}/f_{d}=2$$

The following parameters were selected for the analysis: a sampling frequency of 25,000 Hz, a sample length of 10 s (i.e., 250,000 data points), a spectral resolution of df = 0.1, and a reference pressure of $p_{o}=2\;\cdot 10^{-5}\;Pa$.

**Figure 17:**
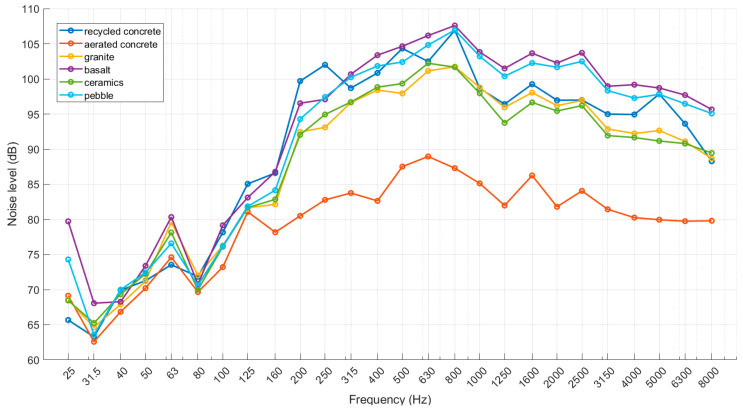
Third-order noise spectrum of the crusher—microphone top position.

**Figure 18:**
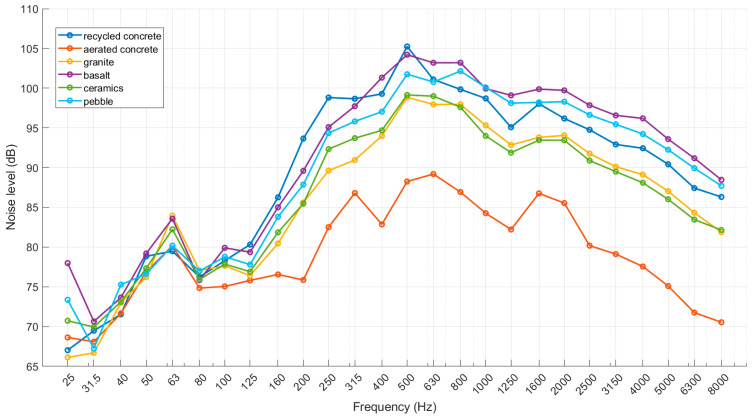
Third-order noise spectrum of a crusher—microphone side view.

**Figure 19:**
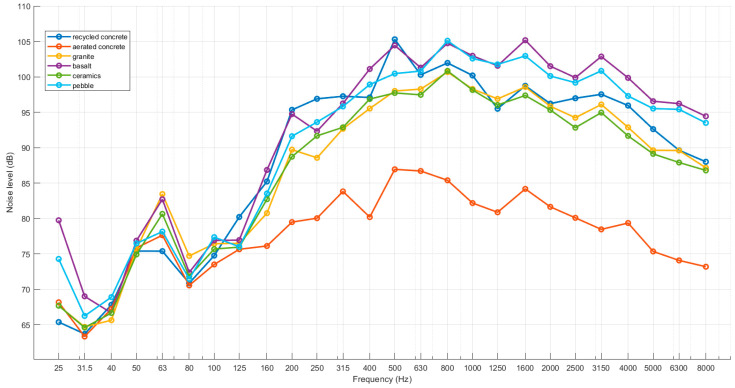
Third-order noise spectrum of a crusher—microphone front position.

Figure 17, Figure 18 and Figure 19 show the sound pressure level in dB. Figure 16 shows the results for the microphone positioned in front of the crusher, Figure 17 shows the results for the microphone positioned to the left of the crusher, while Figure 18 shows the results for the microphone suspended above the crusher, specifically above the feed opening. The first conclusion that comes to mind concerns the sound pressure level—the highest values were recorded by the microphone located in front of the crusher, specifically in front of the stationary jaw. In second place in terms of values is the microphone above the feed opening. The lowest value was recorded on the side of the crusher, where the side wall of the crusher acts as an acoustic barrier. In the subsequent spectra, a structure can be observed that can be preliminarily divided into three characteristic regions:

- low frequencies (6.3–31.5 Hz)—mass motion, kinematic excitation;
- mid frequencies (31.5–250 Hz)—frame and jaw resonances;
- high frequencies (>250 Hz)—material cracking, structural noise.

## 4. Discussion

Although the study was conducted on a laboratory crusher, the controlled experimental conditions allowed for high measurement repeatability and an unambiguous assessment of the influence of the analyzed parameters. Consequently, the main value of this work lies in the identification of relationships and diagnostic features that can serve as the basis for further industrial implementation. However, a final assessment of the method’s usefulness requires validation under actual operating conditions.

It should be noted that this study focuses exclusively on the influence of the feed material on particle size distribution, crushing force, crushing work, the technical efficiency of the crushing process, and noise measurement. The study did not account for variations in other crusher operating parameters, such as shaft rotational speed, CSS discharge gap, and movable jaw stroke.

From the perspective of industrial applications, a more comprehensive analysis should take into account the simultaneous influence of multiple operating parameters and their interactions. Therefore, future research should include the design of multifactorial experiments (e.g., orthogonal tests) and the development of multiparameter models that will enable more accurate prediction and optimization of the crushing process and its operating parameters.

The present study did not analyze the effect of varying feedstock particle sizes for the same material or the material’s moisture content. This limitation was intentional and stemmed from the need to reduce the number of experimental variables so that the influence of the main parameters under analysis on the recorded signals could be clearly assessed. Introducing additional process factors simultaneously, such as grain composition or feed moisture content, could complicate the interpretation of the obtained relationships. It should be emphasized, however, that both particle size and material moisture content can significantly affect the grinding process and the system’s dynamic response; therefore, taking them into account represents a natural direction for further research.

The results obtained indicate varying crushing efficiency for the materials analyzed. The literature emphasizes that crushing efficiency depends on both the feed particle size and the physical and mechanical properties of the material [11], or materials such as river gravel, basalt, and granite, all with the same grain size (16 mm to 22 mm), similar throughput values were obtained, confirming the significant influence of the feed’s geometric parameters.

The decrease in throughput for aerated concrete can be linked to the observed phenomenon of grain jamming in the crusher’s working chamber. This phenomenon is widely described in experimental studies as a factor leading to unstable operation of the equipment and a decrease in its throughput [104].

At the same time, the highest performance was achieved with technical ceramics [86], despite the larger particle size of the feedstock, which indicates the dominant influence of material properties such as brittleness and susceptibility to fracture. Similar relationships have been demonstrated in studies on comminution mechanics, which emphasize that more brittle materials can be effectively comminuted even when the feedstock has a larger particle size [93].

The results obtained thus confirm that evaluating the efficiency of the crushing process requires taking into account both the geometric characteristics of the feed material and its material properties.

The results indicate that the specific energy consumption in the crushing process is relatively low, but clearly depends on the type of material. The observed differences between the tested samples suggest that physico-mechanical properties, such as brittleness, porosity, and internal structure, play a key role, rather than the material type alone. Similar energy values for some materials may result from their similar susceptibility to crack initiation and propagation under impact-compression loading conditions.

The small variation in results may also indicate a limited influence of the feed size itself compared to material properties, which is consistent with trends described in the literature on crushing mechanics. At the same time, it should be noted that under laboratory conditions, the results may be influenced by interfering factors, such as sample heterogeneity or periodic jamming of the material in the crusher’s working chamber.

The results indicate that both total energy consumption and specific energy consumption vary depending on the type of material, with the range of values not being very wide but clearly noticeable. The lowest specific energy consumption was obtained for basalt, while the highest was for cobblestone, suggesting greater resistance of this material to the crushing process.

Similar values for aerated concrete, granite, and ceramic materials indicate that under the applied process conditions, these materials exhibit a similar fracture mechanism and comparable susceptibility to fragmentation. This may result from the compensation of the effects of various material properties, such as porosity, structural heterogeneity, or local brittleness [89].

The relationships obtained are consistent with the literature, which states that specific energy consumption in crushing depends primarily on the material’s physical and mechanical properties—such as compressive strength, internal structure, and susceptibility to crack initiation—rather than solely on feed size or the type of equipment [105]. The literature also emphasizes that energy differences between materials may be relatively small on a laboratory scale, particularly with a limited number of cycles and small samples.

Additionally, it should be noted that some of the observed differences may result from phenomena that disrupt the process, such as periodic jamming of material in the crusher jaws or feed heterogeneity, which affects the instantaneous load on the system and causes a local increase in energy demand [11].

The variation in specific crushing time (1.161–2.544 h/Mg) confirms that the process efficiency is not directly determined by the feed size, but results mainly from the material’s physical and mechanical properties; in particular, the extended time for aerated concrete can be attributed both to its heterogeneous, porous structure, which promotes disruptions in the process, and to its low density, which limits the effective movement of the material within the crusher’s working chamber and its contact with the jaws.

The obtained values of the i_80_ index (2.54–6.56) show significant variation among the analyzed materials; however, their direct comparison is limited due to the use of different initial process conditions. For aerated concrete, recycled concrete, and ceramic materials, a larger feed particle size (d_80_ ≈ 50 mm) was adopted than for the other materials (d_80_ ≈ 20.8 mm), which directly affects the degree of fragmentation.

Consequently, the observed differences in i_80_ are primarily due to the initial conditions of the process, rather than solely to the material properties. This means that this index can be used to evaluate crushing efficiency under homogeneous feed conditions, whereas its application to compare different materials requires normalization or standardization of initial conditions.

The main sources of vibration and noise are the eccentric motion of the shaft, the oscillatory motion of the movable jaw, and the cyclic changes in jaw-material contact forces. Each crushing cycle generates a force impulse that is transmitted to the crusher frame, foundation, and surroundings. Crushing is not a continuous process, but an impact-quasi-static one. The noise generated by this process depends on many factors, including sudden grain fractures, random load distribution, and variable material stiffness in the working gap. This results in a broadband vibration spectrum and randomly occurring peaks of sound pressure changes in the spectrum.

In the future, the authors plan to conduct a detailed analysis of the noise associated with a detailed analysis of the crusher’s vibrations during operation.

It should be emphasized that this study intentionally simplified the experimental conditions by keeping the crusher’s operating parameters constant, such as spindle speed, discharge gap, and feed characteristics. This allowed for an unambiguous assessment of the influence of aggregate type on the crushing process and reduced the variability of the results.

Under actual operating conditions, these parameters may interact with one another, and their combined effects can significantly influence process efficiency. However, the analysis of such interactions is a more complex issue that goes beyond the scope of this study and requires multifactorial research. This issue will be the subject of further research by the authors.

It should be emphasized that this study focuses on the analysis of a single crushing stage under laboratory conditions and is not intended to replicate a complete, multi-stage industrial system. This approach is commonly used in research on comminution processes because it allows for the identification of fundamental mechanisms and a clear assessment of the impact of the analyzed factors. In industrial practice, the aggregate production process is carried out in complex technological systems; however, their modeling and optimization are based on data obtained for individual unit operations. In this context, the presented results provide a basis for further system-level analyses.

## 5. Conclusions

Based on the experiment conducted, it can be concluded that the noise generated by the crushing process depends on the type of aggregate. The graphs showing the change in sound pressure expressed in dB and its level for the tested aggregates correspond to the graphs of forces as a function of the displacement of the movable jaw for the same aggregates. The proposed method of analyzing the noise generated by the crusher is insensitive to transient external disturbances that have lower energy in specific frequency bands. As part of the experiment, the authors formulated the following conclusions:

1. The specific energy consumption ranged from 0.44 to 1.14 kWh/Mg, with the lowest value recorded for basalt and the highest for river gravel.
2. The specific crushing time ranged from 1.16 to 2.54 h/Mg, indicating significant differences in process efficiency, particularly in the case of aerated concrete.
3. The i_80_ index showed considerable variation (2.54–6.56), but its direct comparison across materials is limited due to differences in feed particle size distribution.
4. The results confirm that the efficiency of the crushing process depends primarily on material properties and initial process conditions, such as feed particle size distribution.
5. Based on the results obtained, it can be observed that as aggregate hardness increases, the level of acoustic pressure also increases.
6. As the degree of filling of the crusher chamber increases, the force increases in subsequent operating cycles.

This study fills an identified research gap by providing a comparative analysis of six aggregate types with respect to crushing force, crushing energy, degree of fragmentation, and noise emission. The results expand the available experimental data and provide a broader understanding of the influence of aggregate type on both the mechanical and acoustic characteristics of the crushing process.

A limitation of the study is that it was conducted under controlled laboratory conditions, and the scope of the analysis covered six types of aggregates. Although the results obtained allowed for the identification of differences in crushing parameters and noise emissions, their generalization to other materials and operating conditions requires further research. Future work should include a broader range of materials, an analysis of the impact of additional process parameters, and the validation of results under industrial conditions.

## Figures and Tables

**Table 1:** Summary of research gaps identified in selected studies.

Authors	Research Scope	Main Findings	Identified Gap
Fang et al. [78]	Particle size distribution and energy consumption in crushing	Relationships between energy consumption and particle size distribution were established	Lack of analysis of noise emission in the crushing process
Jeswiet & Szekeres [79]	Energy consumption in comminution processes	Demonstrated the significant contribution of comminution to global energy consumption	Limited consideration of acoustic and environmental aspects
Vasilyeva et al. [80]	Modeling and efficiency improvement of crushing equipment	Identified key factors affecting crusher performance and energy efficiency	Limited consideration of material-dependent behavior and environmental effects
Mazur [81]	Crushing energy and its determinants	Highlighted the large number of variables affecting the crushing process	Lack of clear analysis of individual factors under controlled conditions
Saramak et al. [82]	Dust and noise emission in aggregate production	Identified environmental impacts of crushing operations	Noise treated as a secondary parameter; lack of detailed analysis related to material properties

**Table 2:** Dimensions and weight of feedstock grains.

Materials	Size of Feed Material Grains (mm)	Batch Material Weight (kg)
Pebble	16–22	0.5
Aerated concrete	50	0.5
Basalt	16–22	0.5
Granite	16–22	0.5
Ceramics	50	0.5
Recycled concrete	50	0.5

**Table 3:** The values of examined indicators.

Parameters, Indicators	Pebble	Aerated Concrete	Basalt	Granite	Ceramics	Recycled Concrete
Outlet slot CSS (mm)	5	5	5	5	5	5
Technical performance (Mg/h)	0.60	0.39	0.72	0.77	0.86	0.56
Effective energy (kWh)	0.000570	0.000494	0.000440	0.000494	0.000492	0.000220
Specific energy (kWh/Mg)	1.14	0.988	0.880	0.988	0.984	0.44
Maximum crushing force (kN)	146.54	112.83	112.67	136.24	108.46	25.59
Crushing time (s)	3.00	4.58	2.50	2.33	2.09	3.21
Specific crushing time (h/Mg)	1.667	2.544	1.389	1.294	1.161	1.78
Feed grain size D_80_ (mm)	20.8	50	20.8	20.8	50	50
Product grain size d_80_ (mm)	8.11	7.62	8.18	7.62	7.76	8.20
Degree of fineness i_80_ (-)	2.56	6.56	2.54	2.73	6.44	6.23

## Data Availability

The original contributions presented in this study are included in the article; further inquiries can be directed to the corresponding author.
